# Enhanced Microbial Interactions and Deterministic Successions During Anoxic Decomposition of *Microcystis* Biomass in Lake Sediment

**DOI:** 10.3389/fmicb.2019.02474

**Published:** 2019-10-30

**Authors:** Yu-Fan Wu, Peng Xing, Shuangjiang Liu, Qinglong L. Wu

**Affiliations:** ^1^State Key Laboratory of Microbial Resources, Institute of Microbiology, Chinese Academy of Sciences, Beijing, China; ^2^State Key Laboratory of Lake Science and Environment, Nanjing Institute of Geography & Limnology, Chinese Academy of Sciences, Nanjing, China; ^3^Sino-Danish Centre for Education and Research, University of Chinese Academy of Sciences, Beijing, China; ^4^Technology Center of Zhangjiagang Customs, Zhangjiagang, China

**Keywords:** microbial interaction, deterministic succession, *Microcystis*, anoxic decomposition, *Clostridiaceae*

## Abstract

*Microcystis* biomass remineralization after blooming represents a hotspot of nutrient recycling in eutrophic lakes. Because *Microcystis* blooms are massively deposited on lake sediments, resulting in anoxic conditions, it is important to understand the response and role of benthic microbial communities during the anoxic decomposition of *Microcystis* in freshwater lakes. In the present study, we employed a microcosm method, combined with high-throughput sequencing, functional prediction, and network analysis, to investigate microbial succession during the short-term (30 days) anaerobic decomposition of *Microcystis* in a eutrophic sediment. Continuous accumulation of CH_4_ and CO_2_ and increasing relative abundance of methanogens were observed during the incubation. The microbial community composition (MCC) significantly changed after addition of *Microcystis* biomass, with a shift in the community from a stochastic to a functional, deterministic succession. Families, including *Clostridiaceae*, *Rhodocyclaceae*, *Rikenellaceae*, *Peptostreptococcaceae*, *Syntrophomonadaceae*, *Lachnospiraceae*, and *Methanosarcinaceae*, were predominantly enriched and formed diverse substitution patterns, suggesting a synergistic action of these family members in the decomposition of *Microcystis* biomass. Importantly, intense species-to-species interactions and weak resistance to disturbance were observed in the microbial community after *Microcystis* biomass addition. Collectively, these results suggest that the addition of *Microcystis* induce phylogenetic clustering and structure instability in the sediment microbial community and the synergistic interactions among saprotrophic bacteria play a key role in *Microcystis* biomass remineralization.

## Introduction

Cyanobacterial blooms have become a widespread phenomenon in freshwater habitats (Zhang et al., 2010; Zhai et al., 2013) as one of the harmful consequences of eutrophication, which mainly results from intensive human activities affecting freshwater lakes and reservoirs worldwide (Paerl et al., 2001). As the primary producer, *Cyanobacteria* contribute 0.05% of the global carbon biomass, causing high product accumulation during blooms and making eutrophic lakes extremely active sites for the transport, transformation, and storage of a considerable amount of carbon and nutrients (Wilms et al., 2006). In bloom seasons, high microbial activity leads to oxygen depletion within the uppermost few millimeters of the sediment and even the overlying water (Karlson et al., 2008; Chen et al., 2010). In this scenario, accumulated bloom biomass is primarily mineralized through anaerobic processes in the surface sediment (Bastviken et al., 2008). Furthermore, anaerobic mineralization in the sediments contributes to massive CH_4_ emission from inland water bodies on a global scale (Cole et al., 1994; Schink, 1997; Krinner, 2003; Bastviken et al., 2004a). Thus, it is important to comprehensively understand the microbial transformation process and relevant prerequisite conditions, constituting the basis for the estimation of local and global CH_4_ contribution by anaerobic decomposition of cyanobacterial blooms in inland waters.

Some studies addressing the associated microbial communities during cyanobacterial blooms have been reported, mainly focusing on microbial community dynamics in freshwater environments during the blooms (Li et al., 2012) or bacterial communities associated with certain algae species (Shi et al., 2009). These studies have revealed the influence of algal blooms on the planktonic bacterial community and have also provided important information about the microorganisms involved in algal biomass transformation (Tang et al., 2015).

However, the knowledge of which and how microbes participate in the anaerobic degradation of cyanobacterial blooms in the eutrophic lakes and reservoirs is limited. On the one hand, the involvement of microbial taxa, as well as the interactions between them, are not clear; on the other hand, the limitations of analysis methods have hampered the investigations of the phylogenetic diversity (PD), composition, and dynamics of the microbial community participating in the decomposition of sedimentary algae. In general, the anaerobic decomposition of organic material includes sequential hydrolytic, homoacetogenic, and methanogenic processes (Schink, 1997). Non-representative microbial taxa have been identified for each key step during the anaerobic degradation of cyanobacterial biomass. However, such research has been lagging far behind the analogous research in artificial anaerobic digestion, such as in wastewater treatment plants, where the different types of microorganisms involved have been extensively studied (Li et al., 2014; Narihiro et al., 2015; Shu et al., 2015). Using ^13^C-labeled *Spirulina* biomass and denaturing gradient gel electrophoresis (DGGE), Graue et al. (2012) found that *Psychrilyobacter atlanticus* and some *Propionigenium*-like taxa participated in *Spirulina* biomass anoxic degradation. *In situ Microcystis* scums were incubated for 90 days to simulate the degradation of *Microcystis* biomass in an anoxic water column, and subsequent T-RFLP analysis revealed the predominance of a few novel *Clostridium* spp. during this process (Xing et al., 2011). Shao et al. (2013) investigated the effect of *Microcystis* bloom decomposition on sediment bacterial communities using DGGE in the 100 L mesocosms. The relative abundance of *Bacteroidetes* and *Verrucomicrobia* strongly increased after 14 days. However, it is remarkable that (i) the scenarios of cyanobacterial biomass mineralization in anoxic sediments have been less touched in the available studies; (ii) because of being restricted by the resolution of fingerprinting methods, the important processes in decomposition and the associated microbial groups remain undefined; (iii) greenhouse gas effect of decomposition in the sediment, which is even crucial for the CH_4_ formation in eutrophic aquatic environments, has not been studied well.

The objective of the present study was to determine how sediment microbial communities respond and participate in *Microcystis* anaerobic decomposition after the massive deposition of *Microcystis* into freshwater sediment. Microcosms with sediment slurry and supplemental *Microcystis* biomass under anoxic conditions were generated to examine the sediment microbial communities after incubation for 30 days. Illumina MiSeq sequencing was applied to the sequential samples to elucidate the differential patterns with high taxonomic resolution. To further depict the inherent response of the microbial community to *Microcystis* addition, the relationship among co-occurring species was evaluated, and the community function profiles were also predicted based on the structural data. We observed distinct microbial succession during the anoxic decomposition of *Microcystis* in freshwater sediment, characterized by enhanced species-species interaction and low resistance to disturbance. Such works are helpful for a better understanding of algae biomass transformation and greenhouse gas production, constituting the center of carbon cycling in eutrophic lakes.

## Materials and Methods

### Microcosm Setup

The *Microcystis* strain used was *Microcystis* sp. FACH-B 7806 (obtained from Freshwater Algae Culture Collection at the Institute of Hydrobiology, Chinese Academy of Sciences). *Microcystis* biomass was collected in the lab, cultivated in BG11 medium^1^ under a luminance of 1200 Lux and 12 h dark: 12 h light photoperiod for 15 days, then collected by centrifugation and dried using a freeze dryer (ALPHA 1-2, CHRIST, Osterode am Harz, Germany). The *Microcystis* powder was UV sterilized for 20 min and stored at −80°C before adding to the microcosms. The sediment slurry used for the anaerobic *Microcystis* biodegradation tests were collected from Meiliang Bay (31.28946° N, 120.812905° E) of Lake Taihu in October 2012. Prior to the experiment, the sediments (1–5 cm deep) were sieved using a 0.6-mm mesh to remove the macrofauna and debris and subsequently incubated at room temperature (20–25°C) for 2 weeks to decrease the organic carbon content.

More than 60 microcosms built up in the sterile 100-mL air-tight glass bottles (100-mL capacity; CNW Technologies GmbH, Düsseldorf, Germany), which were divided into two groups. Each bottle in the control group contained 12.5 g homogenized sediment slurry +10 mL of sterile water, and in the treatment group, 12.5 g homogenized sediment slurry +10 mL of sterile water +25 mg freeze-dried *Microcystis* biomass. All the bottles were sealed with rubber stoppers and aluminum seals and wrapped with a silver paper to keep them in the dark. The headspace in the bottles was vacuumed for 10 s and filled with purified N_2_ gas four times to maintain the anoxic conditions. All the microcosms were incubated in an incubator at 25°C.

### Sampling and Physicochemical Analyses

During the incubation, six bottles from the treatment group and two bottles from the control group were randomly selected at each time point of (0, 1, 3, 10, 17, 23, and 30) days. The headspace in each jar was sampled with a 500 μL syringe (Part Number/REF: 81256, HAMILTON, Romania) after vigorously shaking to remove the gas bubbles trapped in the sediment slurry. The concentrations of H_2_, CO_2_, and CH_4_ in the headspace were measured on a gas chromatograph equipped with a packed column TDX-01 (Techcomp, Shanghai, China), a flame ionization detector (FID), and a thermal conductivity detector (TCD). The gas concentration was calibrated using H_2_, CH_4_, and CO_2_ standards purchased from the National Institute of Metrology P. R. China Standard Gas Testing Center. The bottles were sacrificed after gas measurement. A pH meter (METTLER TOLEDO FE20, Shanghai, China) was used to measure the pH of the slurry.

### Microbial Biomass Collection and DNA Extraction

The sediment slurry samples were collected and stored at −20°C after freeze-drying. Genomic DNA was extracted from 0.25 g of freeze-dried sediment slurry using the FastDNA SPIN Kit (MP Biomedicals, Santa Ana, CA, United States) according to the manufacturer’s instructions. The genomic DNA was quantified using a spectrophotometer (GE, Pittsburgh, United States) and stored at −20°C until subsequent procedures.

### Real-Time Quantitative PCR

The 16S rRNA genes of the total bacteria were quantified by real-time PCR using the primer set 341F and 534R (Bru et al., 2008). Total Archaea were quantified using the 16S rRNA gene primer set Arch 333F and Arch 554R (Suzuki et al., 2000). Methanogens were quantified using the primer set ME1 and mlas (Steinberg and Regan, 2008), which targets the *mcrA* gene. The PCR conditions were as described by Nunoura et al. (2006). To construct standard curves for quantitative PCR, the PCR products for the partial bacterial 16S rRNA (341F and 534R), archaeal 16S rRNA (Arch333F and Arch554R), and *mcrA* (ME1 and mlas) genes (Supplementary Table S1) were purified and cloned into the pMD18-T vector (Takara, Dalian, China), and subsequently transformed into *Escherichia coli* DH5α competent cells (Takara, Dalian, China). The successfully inserted plasmids were extracted using a Plasmid Mini-Prep Kit (Axygen Biosciences, Union City, CA, United States), and the concentrations were determined by spectrophotometry using a NanoVue (GE, Pittsburgh, United States). The standard curves were prepared from linearized plasmid serial dilutions of 10^0^ to 10^8^ gene copies directly calculated from the concentration of the extracted plasmid. Quantitative PCR was performed in a 20 μL reaction comprising 10 μL of SYBR Premix ExTaq^TM^ (Takara, Dalian, China), 0.25 μM each primer, 1 μL of 1/10 diluted DNA, and RNase-free water. The thermocycling program for quantitative PCR comprised an initial cycle at 95°C for 30 s, followed by 40 cycles at 95°C for 5 s and 60°C for 34 s. All measurements were obtained in triplicate. The standard curves were used as the references to calculate the copy number of bacterial 16S rRNA, archaeal 16S rRNA, and methanogens *mcrA* genes (correlation coefficient *r*^2^ = 0.993, 0.993, and 0.993, respectively). The efficiencies of the qPCR amplification for the bacteria, archaea, and *mcrA* were 105.8, 96.6, and 97.1%, respectively.

### Illumina MiSeq Sequencing and Data Processing

Genomic DNA was extracted from six replicates in the treatment group and two replicates in the control group at (0, 1, 3, 10, 17, 23, and 30) days. To analyze the bacterial diversity during cultivation, the V4-V6 region of the bacterial 16S rRNA genes was amplified and sequenced on an Illumina MiSeq platform. The V4-V6 region of the 16S rRNA gene was amplified using the primers 515F (5′- GTGCCAGCMGCCGCGGTAA-3′) and 907R (5′- CCGTCAATTCCTTTRAGTTT-3′) (Zhou X. et al., 2015). PCR amplification was performed using an ABI GeneAmp^®^ 9700 thermal cycler (Applied Biosystems, Waltham, MA, United States). The PCR reaction was performed in a 20-μL reaction volume containing 10 μL of Trans Start Fast pfu DNA Polymerase Super Mix (Trans Gen AP221-02), 0.2 μM of forward and reverse primers, and 10 ng of template DNA. The thermal cycling parameters were denaturation at 95°C for 1 min, followed by 30 cycles at 94°C for 10 s, 55°C for 30 s, and 72°C for 45 s, with a final step at 72°C for 5 min. The PCR products were analyzed on a 2% agarose gel, purified using the AxyPrep^TM^ DNA Gel Extraction Kit (Axygen Biosciences, Union City, CA, United States) according to the manufacturer’s instructions, and quantified using QuantiFluor^TM^ -ST (Promega, Fitchburg, WI, United States). The purified amplicons were pooled in equal molar ratios and paired-end sequenced (2 × 250 bp) on an Illumina MiSeq platform according to the standard protocols of the Major Bio Co., Ltd. (Shanghai, China).

The raw sequencing reads were de-multiplexed, quality-filtered, and analyzed using QIIME v1.17 (Caporaso et al., 2010). Sequence quality management and operational taxonomic units (OTUs) analysis were conducted using the UPARSE pipeline according to Edgar (2013). Briefly, reads <250 bp, with an average quality score (Q score) of <25 in a sliding window of 50 bp, with mismatched primer sequences, or containing ambiguous bases (Ns) were removed from downstream analyses. Both the forward and reverse primers were truncated from the reads. Chimeric sequences were removed using USEARCH software (Edgar, 2010) based on the UCHIME algorithm (Edgar et al., 2011). After quality and chimera filtering, 1.73 million reads with an average read length of 395 bp in 56 samples were obtained. The lowest number of sequences was 13,628. Therefore, the sequences in the samples were randomly normalized to 13,628 prior to conducting subsequent analyses. All the reads were clustered into OTUs at 97% pairwise identity using UCLUST (Edgar, 2013). Representative OTUs were aligned to the Greengenes database (gg_13_8_otus).

### Community Diversity Analysis and Null Model Test

Alpha diversity was estimated according to the Shannon index, Chao1, observed species, and PD for all the samples by using QIIME v1.17. Beta diversity was based on Bray–Curtis algorithm of the OTU table to identify the clustering patterns in the microbial communities among the samples across the incubation times (R-vegan function vegdist). In addition, the null model Raup-Crick index (β_RC_) was used to assess whether the null-expected number of shared species between any two communities was different from the observed number of shared species (Chase et al., 2011). The null community was generated by randomly shuffling the original community 199 times with the independent swap algorithm by holding the number of OTUs in each sample and the number of samples, in which each OTU appears constant. The PAST program (Hammer et al., 2001) was used for these analyses. Both the Bray–Curtis and Raup-Crick algorithms were visualized on the 2D non-metric multidimensional scaling (NMDS) plots using the program PRIMER v7 (Clarke and Gorley, 2015).

### Microbial Functional Prediction and Network Construction

The functional profiles of the microbial communities were predicted by using PICRUSt (Phylogenetic Investigation of Communities by Reconstruction of Unobserved States^2^; Langille et al., 2013) based on our 16S rRNA data. The 6,763 close-reference OTUs were picked up with Greengenes 13.5 in Galaxy^3^ and their functional profiles were predicted from the Kyoto Encyclopedia of Genes and Genomes (KEGG) pathways (Kanehisa et al., 2014). The average nearest sequenced taxon index (NSTI, 0.182 ± 0.02 SD) of these samples was near that reported for soil communities (0.17 ± 0.02) (Langille et al., 2013). To identify functional and metabolic subsystems, whose relative frequencies (rel. freq. %) in treatments differed significantly from that in control samples based on KEGG, two-sided Welch’s *t*-test (*p*-value) was used in the STAMP v2.01 software (Parks et al., 2014).

Potential interactions between co-occurring microbial taxa in control and treatment were determined by molecular ecological networks (MENs), which were constructed by using a random matrix theory (RMT)-based approach on an open-accessible comprehensive pipeline (Molecular Ecological Network Analysis Pipeline, MENAP^4^, Deng et al., 2012). In the construction of the MENs, data standardization, Spearman’s correlation estimation, adjacency matrix determination by RMT-based approach, network characterization, module detection, eigengene network analysis, and network comparisons were accomplished in order (Zhou et al., 2010). For each network, 100 corresponding random networks were then generated with the same network size and an average number of links. A Z-test was applied to determine the differences in the indices between the constructed and random networks. Cytoscape 3.4.0 was used for the network visualization.

### Statistical Analysis

One-way ANOVA was used to identify the statistical significance of the pH value within each group along the incubation time. The differences of alpha diversity index, as well as the q-PCR abundance of bacteria, archaea, and methanogens, between the control and treatment groups was evaluated by Independent-Samples *t-*test (SPSS v20.0) at a single time point. Heatmaps representing the relative sequence abundances of bacterial OTUs among the samples were constructed using the “pheatmap” and “gplots” packages. Significantly enriched microbial taxa during the treatment was evaluated by Independent-Samples *t*-test (SPSS v20.0) at each time point. The relationships between relative abundance and incubation time for each group were then explored with four models, linear, quadratic, exponential, and logarithmic regression. The best models were selected based on Akaike’s information criterion (AIC, Bozdogan, 1987). Permutational multivariate analysis of variance (PERMANOVA) (Anderson, 2001) was performed using the adonis function in the Vegan package (R studio, version 2.14.1, R Development Core Team, 2011) to evaluate the two-sided effects of *Microcystis* addition and incubation time on microbial community dynamics. The correlation between methane concentration and methanogen abundance was measured by Pearson correlation coefficient (*r*-value, SPSS v20.0).

### Deposition of DNA Sequences

All the DNA sequences retrieved from the control and treatment groups at different sampling times were deposited at the European Nucleotide Archive and can be found under the Accession number PRJEB21014^5^.

## Results

### Decomposition of *Microcystis* Biomass and CH_4_/CO_2_ Production

**FIGURE 1:**
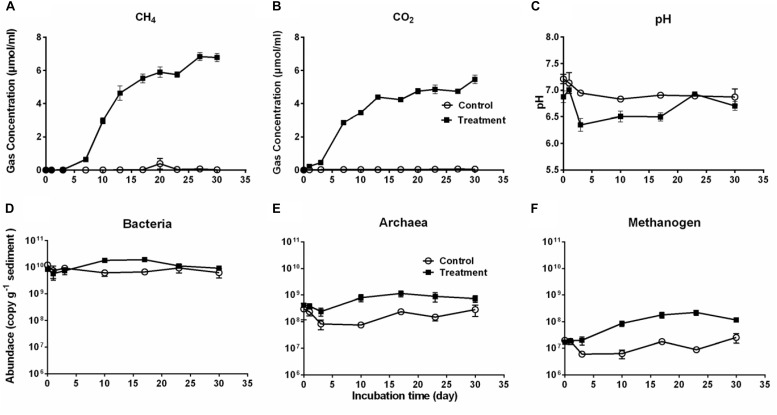
The cumulative CH_4_ **(A)** and CO_2_ **(B)** production, pH **(C)** in the microcosms, and the abundances of Bacteria **(D)**, Archaea **(E)**, and Methanogens **(F)** during 30-day cultivation in the microcosm without (Control, open circles) or with (Treatment, solid squares) *Microcystis* addition. Microbial abundances were determined by qPCR method.

Production of CO_2_ and CH_4_ is an indicator of *Microcystis* decomposition, and they continuously accumulated after *Microcystis* addition, with final concentrations of 27.10 ± 2.28 μmol cm^–3^ sediment and 21.87 ± 2.29 μmol cm^–3^ sediment, respectively (Figures 1A,B). The CH_4_ production exhibited a sigmoidal curve with a lag phase (from day 0 to day 7), an exponential phase (from day 7 to day 17), and a stationary phase (from day 23 to day 30). CO_2_ rapidly accumulated from the beginning to the stationary phase (day 17) and remained stable throughout the experiment. In the control group, the production of CO_2_ and CH_4_ was not detectable. The addition of *Microcystis* biomass caused no significant pH difference in the treatment group (one-way ANOVA, *p* > 0.5), which seemed more stable (6.66–7.06) than the control (6.19–7.30) throughout the 30-day period (Figure 1C).

Accordingly, the bacterial abundance increased in the treatment group from day 1 to day 17, followed by a slight decrease, whereas it remained stable in the control group (Figure 1D). The abundance of Archaea and methanogens were significantly higher in the treatment group than in the control group (for both, Independent-Samples *t*-test, *p* = 0.001) (Figures 1E,F). As a subgroup of the total archaeal population, methanogens accounted for approximately 2–47% of the total Archaea. There was a close correlation between the abundance of methanogens and CH_4_ production in the treatment group (Pearson’s correlation *r* = 0.88, *p* = 0.009) but not in the control group (Pearson’s correlation *r* = −0.04, *p* = 0.931).

### Microbial Community Diversity

In total, 18,540 OTUs were detected and classified into 73 phyla and 1,011 genera. We observed significantly lower OTU numbers, Shannon index, and PD value in the treatment group than in the control group (Table 1). For the Chao1 estimates of microbial communities component, the treatment group was significantly lower than the control during the first 10 days of the incubation and then no significant difference was observed between the two groups (Table 1).

**TABLE 1:** Comparison of microbial community diversity between microcosms without (Control) or with *Microcystis* (Treatment) addition during the incubation course.

	**Observed OTUs**		**Shannon index**		**Chao1**		**PD**	
**Day**	**Control**	**Treatment**	**Control**	**Treatment**	**Control**	**Treatment**	**Control**	**Treatment**
0	5654(±808)	5277(±420)	10.41(±0.05)	9.42(±0.31)	9571.52(±1499.6)	9420.07(±1563.9)	255.9(±25.9)	246.8(±18.37)
1	5150(±110)	2242(±245)^∗∗^	10.33(±0.001)	6.79(±0.46)^∗∗^	9084.26(±47.2)	4524.54(±143.5)^∗∗^	243.0(±4.5)	128.7(±9.2)^∗∗^
3	5324(±823)	2435(±607)^∗∗^	10.30(±0.16)	6.79(±0.67)^∗∗^	8739.55(±2068.8)	4631.02(±960.2)^∗^	248.2(±30.7)	135.9(±25.5)^∗∗^
10	5698(±814)	2717(±368)^∗∗^	10.31(±0.05)	7.22(±0.58)^∗∗^	11037.34(±2743.7)	5489.47(±736.8)^∗∗^	259.9(±27.1)	150.7(±15.4)^∗∗^
17	4722(±95)	3239(±509)^∗^	10.13(±0.05)	7.76(±0.35)^∗^	7656.56(±343.8)	6261.16(±1035.9)	223.9(±5.2)	168.7(±20.7)^∗^
23	4917(±690)	3021(±497)^∗^	10.36(±0.09)	8.26(±0.33)^∗^	8028.89(±743.8)	5284.65(±1164.6)	231.7(±26.5)	161.6(±20.2)^∗^
30	4644(±314)	2823(±464)^∗^	10.13(±0.001)	8.11(±0.44)^∗^	7695.61(±299.5)	5088.86(±809.4)	219.2(±11.9)	152.7(±18.6)^∗^

*All data are listed as: mean (±SD). The statistical significance of Control and Treatment group was evaluated by the Independent-Samples *t*-test and statistical levels are indicated by asterisks of *p* ≤ 0.01 (two asterisks, ^∗∗^), *p* ≤ 0.05 (one asterisk, ^∗^) and *p* > 0.05 (no asterisk).*

**FIGURE 2:**
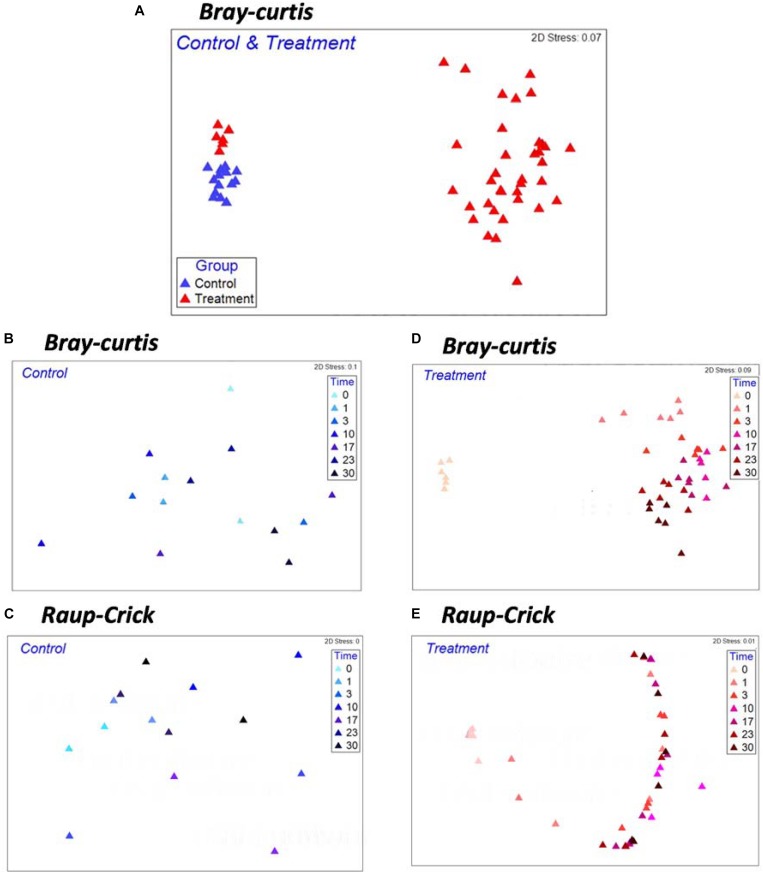
Non-metric multi-dimensional scaling (NMDS) ordinations based on Bray–Curtis Index **(A,B,D)** and β_RC_ **(C,E)** for Control and Treatment examining the effects of *Microcystis* biomass addition on patterns of microbial community compositions. Each symbol in the NMDS plot represents a sample collected during the incubation.

Non-metric multidimensional scaling analysis with whole samples revealed a clear separation of the microbial community composition (MCC) between the control and treatment groups (Figure 2A). The MCC in the treatment group significantly changed after *Microcystis* biomass addition (Figure 2D and Supplementary Figure S1, PERMANOVA, *F*_1_,_42_ = 17.02, *p* < 0.001), whereas in the control group, the MCC remained almost undisturbed (Figure 2B). Random sampling effects were primarily observed in the control samples during incubation, and there was no difference in β_RC_ before and after the incubation course (Figures 2B,C). However, the β_RC_ values of the treatment samples indicated that the MCC after *Microcystis* biomass addition was more similar to the start-point samples than expected by chance (Figures 2D,E). This observation suggests that the changes in β-diversity did not reflect the random influence of incubation on the microbial species and that the input organic matter provided a systematic ecological filter for the removal of some species from each community exposed to the substrate.

### Microbial Succession During *Microcystis* Degradation

**FIGURE 3:**
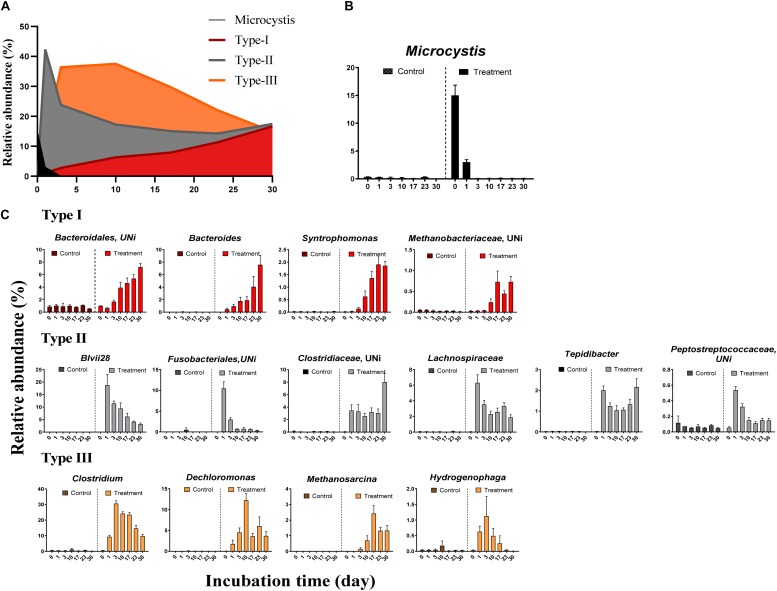
The relative abundance of *Microcystis* and three distinct microbial groups during the incubation. **(A)** The overview of relative abundance of *Microcystis* and three bacterial groups in Treatment samples; **(B)** the decay of *Microcystis* along the time; **(C)** variation of the relative abundance for representative genera in each group. The colors in subfigure of **(C)** are corresponding to those in panel **(A)**.

We observed systematic successions of the relative abundances of dominant OTUs (Figure 3A) during *Microcystis* degradation (Figure 3B). Based on the AIC test, microbial successions can be classified into three distinct types (Figure 3C and Table 2). (i) The type I group exhibited an exponential increase in abundance across time. Representative taxa included *Bacteroides*, *Syntrophomonas*, and unidentified genera from *Bacteroidales* and *Methanobacteriaceae*. (ii) The type II group exhibited a quadratic regression along time with positive binomial coefficients. Representative taxa included *Tepidibacter*, unidentified *Fusobacteriales*, *Lachnospiriaceae* and unidentified genera from *Clostridiaceae*. The relative abundance sharply increased after the addition of *Microcystis* and remained relatively stable during the incubation. (iii) The type III group exhibited a quadratic regression along time with negative binomial coefficients. Representative taxa included *Clostridium*, *Dechloromonas*, *Methanosarcina*, and *Hydrogenophaga*.

**TABLE 2:** The dynamics of the significantly enriched groups were simulated by the multiple regression models.

**Group**	**Best models^a^**	***r*^2^**	***p*-value**	**Equation**	**Constants**
*Bacteroidales*	Exponential/linear/quadric	0.69	<0.001	Type I: Exponential *y* = *a* × *e*^*T* ± *b*	*a*:0.21, *b*:0.79
*Bacteroides*	Exponential/quadric	0.47	<0.001		*a*:0.22, *b*:−0.44
*Methanobacteriaceae*	Exponential/linear/quadric	0.40	<0.001		*a*:0.02, *b*:0.00
*Syntrophomonas*	Exponential/quadric	0.68	<0.001		*a*:0.07, *b*:−0.08
*Clostridiaceae*	Quadric/exponential	0.25	<0.01	Type II: Quadric *y* = *aT*^2^ ± *bT* ± *c*	*a*:1.71, *b*:−6.28, *c*:7.59
*Tepidibacter*	Quadric	0.36	<0.001		*a*:0.60, *b*:−2.54, *c*:3.53
*Blvii28*	Logarithmic/linear/quadric	0.53	<0.001		*a*:0.57, *b*:−7.56, *c*:22.83
*Fusobacteriales*	Quadric	0.81	<0.001		*a*:2.36, *b*:−12.96, *c*:17.68
*Lachnospiraceae*	Quadric/logarithmic	0.48	<0.001		*a*:0.86, *b*:−4.79, *c*:8.97
*Peptostreptococcaceae*	Quadric	0.80	<0.001		*a*:0.09, *b*:−0.49, *c*:0.84
*Clostridium*	Quadric	0.74	<0.001	Type III: Quadric *y* = *aT*^2^ ± *bT* ± *c*	*a*:−10.72, *b*:43.23, *c*:−13.44
*Dechloromonas*	Quadric	0.30	<0.01		*a*:−3.43, *b*:15.03, *c*:−7.73
*Hydrogenophaga*	Exponential/linear/quadric	0.21	<0.05		*a*:−0.34, *b*:0.67, *c*:−0.40
*Methanosarcina*	Linear/logarithmic/quadric	0.39	<0.001		*a*:−0.08, *b*:0.99, *c*:−0.81

*^*a*^The best models were selected based on the minimum Akaike’s Information Criterion (AIC) value. If the difference of AIC value between two models <2, there is no difference in their performance. *T* in the equations means incubation time (days).*

**FIGURE 4:**
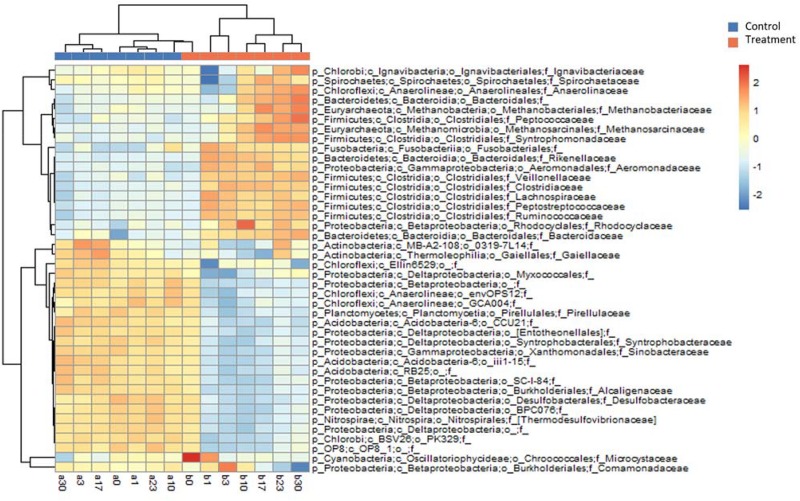
Heatmap diagram showing relative abundances of the top 25 families selected from Control and Treatment (a total of 42 families). Upper dendrogram shows community similarity among samples and left dendrogram depicts clustering of families by co-occurrence.

The differences in the MCC between the control and treatment groups were also significant at coarse taxonomic resolution. For example, at the phylum level, the relative abundances of the abundant phyla in the control group remained stable throughout the experiment. However, in the treatment group, the relative abundances of *Firmicutes* and *Bacteroidetes* immediately increased after *Microcystis* addition, whereas the relative abundances of *Proteobacteria* and *Cyanobacteria* (*Microcystis*) rapidly decreased (Supplementary Figure S2). At the family level, eight families in the treatment group, *Clostridiaceae*, *Lachnospiraceae*, *Peptostreptococcaceae*, *Ruminococcaceae*, *Veillonellaceae*, *Bacteroidaceae*, *Rikenellaceae*, and *Aeromonadaceae* immediately increased in abundance after *Microcystis* addition (Figure 4). Interestingly, five of these families belong to the same order of *Clostridiales*. The relative abundance of methanogens also steadily increased from 0.8% on day 0 to 3.8% on day 17 and remained stable thereafter (Supplementary Figure S3). In addition to the increasing abundance of methanogens, the families *Anaerolinaceae*, *Spirochaetaceae*, *Peptococcaceae*, and *Syntrophomonadaceae* also increased in abundance on day 17 (Figure 4).

### Network Analyses and Community Functional Prediction

**FIGURE 5:**
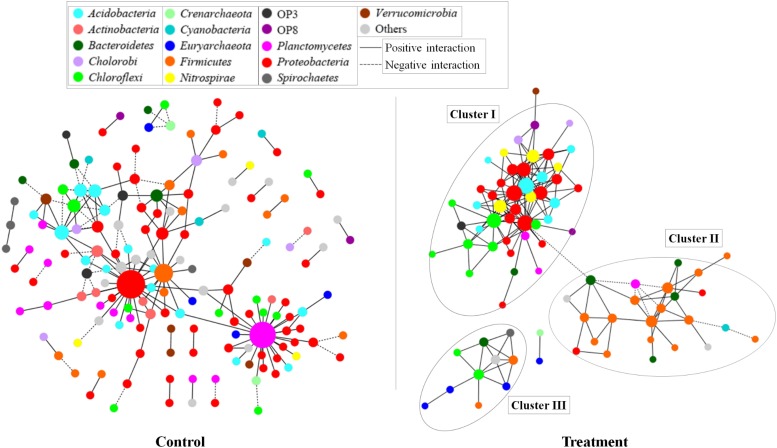
Networks based on correlation analysis of co-occurring bacterial and archaeal genera in a 30-day incubation with (treatment) or without (control) *Microcystis* addition. A connection between nodes stands for a statistically significant positive (a Spearman’s ρ > 0.8 and *p* < 0.01, red line) or negative (b Spearman’s ρ < -0.8 and *p* < 0.01, blue line) correlation. The size of each node is proportional to the number of connections (that is, degree). The nodes were colored by taxonomy, the top 20 abundant phyla are shown in different colors and all the others were in gray.

Based on the network analysis, 157 and 82 nodes were obtained from the control and Treatment, respectively. Positive correlations were predominant in both networks with rare negative correlations (Figure 5). The clustering coefficients and harmonic geodesic distance were significantly different from those of the corresponding random networks with the same network size and average number of links (Table 3), indicating that the MENs in both the control and the treatment showed small-world characteristics. In comparison with the control, MENs in the treatment generally had significantly higher connectivity, higher clustering efficiencies, and fewer modules (Table 3). Focusing on the microbial associations within the treatment group showed that three clusters were distinct in the treatment network (Figure 5). Cluster I, including OTUs affiliated with the phyla *Proteobacteria-Acidobacteria-Chloroflexi*, showed co-occurrence among most nodes within it, and Cluster II, including the phyla *Bacteroidetes-Firmicutes*, showed an overall negative connection with Cluster I. Furthermore, *Microcystis* formed a co-exclusive pattern with other bacterial taxa in Cluster II, and Cluster III, including methanogens, *Syntrophomonas* and *Longilinea*, was independent of Cluster I and II.

**TABLE 3:** Topological properties of the empirical molecular ecological networks (MENs) for the control and treatment group and their associated random MENs.

	**Group**	**Control**	**Treatment**
Empirical networks	Similarity threshold (St)	0.86	0.86
	Network size (*n*)	157	82
	*R*^2^ of power law	0.873	0.788
	Harmonic geodesic distance (HD)	2.127	2.768
	Average connectivity (avgK)	2.328	4.561
	Average clustering coefficient (avgCC)	0.127	0.373
	Modularity	0.729 (24)	0.581 (6)
	Transitivity (Trans)	0.105	0.36
Random networks	Harmonic geodesic distance (HD ± SD)	2.557 ± 0.262	2.915 ± 0.127
	Average clustering coefficient (avgCC ± SD)	0.042 ± 0.014	0.101 ± 0.019
	Modularity ± SD	0.646 ± 0.012	0.384 ± 0.012

**FIGURE 6:**
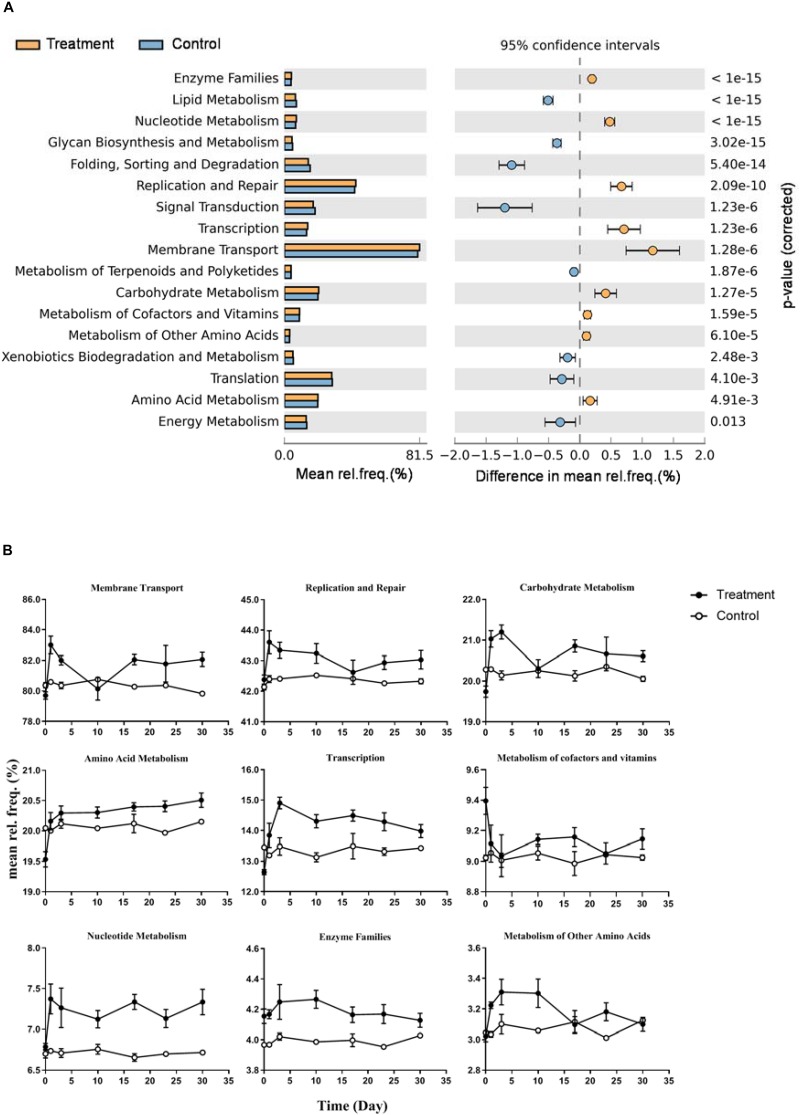
Differences of predicted microbial community function between Control and Treatment throughout the incubation. **(A)** KEGG subpathway I with significant different relative frequency (rel. freq. %) picked up by STAMP with a two-sided Welch’s *t*-test of a symptomatic confidence intervals (0.95). Subpathways overrepresented in the Treatment which have a positive value of proportions are indicated by orange filled circles and those overrepresented in the Control are indicated by blue filled circles. **(B)** Nine line charts showing specific dynamics of the overrepresented pathways in Treatment group (corresponding to orange filled circles in panel **A**) during the incubation.

Functional prediction indicated that17 subpathways I belong to four major categories were significantly different between control and treatment (Figure 6A, *p* < 0.01). Nine of them were significantly enriched in the microcosms of the treatment group, including membrane transport, replication and repair, carbohydrate metabolism, amino acid metabolism, transcription, metabolism of cofactors and vitamins, nucleotide metabolism, enzyme families, and metabolism of other amino acids. The dynamics of relative frequencies along time are shown in Figure 6B. Five of the nine subpathways I belonged to the category metabolism. In subpathway II, we observed increased frequency of genes distributed in diverse pathways in the treatment group (Supplementary Figure S4). For example, within the carbohydrate metabolism, six metabolic pathways showed higher frequency in the treatment samples: fructose and mannose metabolism, galactose metabolism, starch and sucrose metabolism, the pentose phosphate pathway, pentose and glucuronate interconversions, and amino sugar and nucleotide sugar metabolism.

## Discussion

### Efficient Production of CH_4_ From Anoxic Decomposition of *Microcystis*

Blooming *Microcystis* form scum, which is massively deposited onto lake sediments, resulting in rapid oxygen depletion on the surface sediment (Xing et al., 2011). A positive correlation between the biomass of *Microcystis* and CH_4_ production has been observed in the littoral zones of hypereutrophic Lake Taihu, suggesting that the biomass of *Microcystis* is actually transformed to CH_4_ in the field (Wang et al., 2006). The observations of this study further demonstrated that sedimentary *Microcystis* blooms could enhance the accumulation of CO_2_ and CH_4_ in the anaerobic sediment, and this process occurred instantly after *Microcystis* biomass addition.

In the present study, approximately 14.56 mL of CH_4_ (equivalent to 7.8 mg carbon, estimated with the gas density of 0.717 g/L at standard condition) was produced from the initial 25 mg dry-weight of *Microcystis* biomass (equivalent to 9.5 mg carbon with chemical formula C_106_H_263_O_110_N_16_P, molecular weight 3350 g/mol) during the 30-day anaerobic incubation, indicating an efficient transformation from algal organic carbon to greenhouse gases (∼82.5% conversion efficiency). Based on water content of 99% in living *Microcystis* cells, 1 kg of wet algal biomass could be degraded with ∼5.82 L of CH_4_ gas production. In Lake Taihu, cyanobacterial blooms might reproduce at a rate of at least 0.25 million kilograms (weight wet) per year during the whole bloom season (personal communication with Dr. Hongtao Duan). In this case, 1.46 million liters of CH_4_ (equivalent to 1043 kg) would be generated under anoxic or anaerobic conditions. Please note that this estimate does not take into account the contribution of other organic matter in sediments to the methane formation under anoxic and anaerobic conditions. The real lake ecosystems are much more complex and dynamic than the microcosms built up in this study; therefore, the conversion efficiency from cyanobacterial biomass to CH_4_ needs to be further evaluated before its application. In any case, methane accounts for approximately 20% of the greenhouse effect (Wuebbles and Hayhoe, 2002; Sessions et al., 2009), significantly contributing to global warming. The present study evidently demonstrates that, under hypertrophic conditions, accumulated CH_4_ induced by cyanobacterial blooms is an important component of methane budgets in local lakes as well as in the drainage basin (Bastviken et al., 2004b, 2008, 2011).

### Distinct Microbial Succession During Anaerobic Degradation of *Microcystis*

Although decomposition of organic material under anaerobic conditions is generally assumed to include hydrolysis, acidogenesis, syntrophic acetogenesis, and methanogenesis (Schink, 2002), the composition and dynamics of the microbial communities involved in *Microcystis* biodegradation is far from well-documented. In the present study, we investigated the MCC based on Illumina Miseq sequencing, enabling the detection of the distinct pattern of microbial successions during *Microcystis* degradation.

During the hydrolysis phase, *Bacteroides* spp. and an unidentified genus in *Bacteroidales* continuously increased in abundance (type I group). The superb ability of these bacteria for degradation of polymers, particularly various types of polysaccharides (Wexler, 2007; Ravcheev et al., 2013), initiated flourishing during *Microcystis* degradation. The bacteria taxa belong to type II, such as the *Blvii28* group (*Rikenellaceae*), *Fusobacteriales, Lachnospiraceae*, and *Peptostreptococcaceae*, rapidly responded to *Microcystis* biomass addition and peaked at Day 1. Most of them were considered as hydrolytic fermenting bacteria in the degradation of complex polysaccharides (Tholozan et al., 1992; Bennett and Eley, 1993; Schink, 2002; Zhou L. et al., 2015). Some of the 16S rRNA sequences from *Blvii28* were highly similar (∼99% identity) with that of *Acetobacteroides hydrogenigenes*, which is involved in the conversion of carbohydrates to acetate and CO_2_ (Su et al., 2014). *Clostridium* and *Dechloromonas* from the type III group also strongly increased in abundance during the initial days of incubation. Bacteria in the genus *Clostridium* are commonly known as hydrolytic (saccharolytic and proteolytic) and fermentative bacteria that can ferment various polysaccharides to volatile fatty acids, H_2_, and CO_2_ (Ruan et al., 2014; Deng et al., 2015). The importance of *Clostridium* species during the anaerobic degradation of organic material in freshwater environments has previously been identified (Mallet et al., 2004; Xing et al., 2011), and *Dechloromonas* is frequently detected in various degradation and nitrate reduction processes of monoaromatic compounds (Coates et al., 2001).

The increase in the abundance of syntrophic bacteria after Day 10 reflected the accumulation of fatty acid intermediates during *Microcystis* biomass decomposition. Syntrophic acetogens (in type I group), such as *Syntrophomonas*, was significantly promoted during *Microcystis* degradation, suggesting that these species are more likely to utilize the intermediates generated from *Microcystis* hydrolysis. *Syntrophomonas* species utilize a variety of fatty acids ranging from C4 to C8 or other longer fatty acids and coexist with butyrate-producing bacteria (McInerney et al., 1981, 2008).

Methanogens, such as *Methanobacteriaceae* and *Methanosarcina* species, were significantly enriched during the degradation. The most dominant *Methanosarcina* reached the highest abundance on day 17. Species within *Methanosarcina* can use all the methanogenic substrates, H_2_ and CO_2_, methanol, methylamine, methyl sulfides, and acetate, except for format (Galagan et al., 2002; Lambie et al., 2015). The increase in the abundance of these methanogens suggested that diverse substrates were available for CH_4_ synthesis.

Overall, hydrolytic-fermenting bacteria (such as *Blvii28* group, *Clostridium*, and *Bacteroidales* species), syntrophic acetogens (*Syntrophomonas*), and methanogens (*Methanobacteriaceae* and *Methanosarcina*) were significantly enriched, and their succession patterns suggested that these microbial groups might possess unique substrate utilization preferences and could synergistically function during the degradation of *Microcystis*.

### Predicted Community Function and Inter-Specific Connections

Studies have shown that ecosystem properties greatly depend on biodiversity due to differences in the functional characteristics of organisms present in the ecosystem (Bell et al., 2005; Hooper et al., 2005). Based on diversity-stability relationships (Naeem and Li, 1997), the lower α-diversity (Shannon and PD index) in the treatment group indicated a weaker anti-interference ability of the microbial community during *Microcystis* degradation. Moreover, good consistency was found between the realistic Bray–Curtis dissimilarity and the probabilistic β_RC_ index for the treatment, which suggested the relative importance of deterministic (niche-related) processes in their microbial community assembly. Therefore, the input of organic matter imposed as a systematic ecological filtering to enhance partial members with specific functional characteristics in the community (Zhang et al., 2018).

During the anaerobic decomposition of *Microcystis*, the relative frequency and dominance of metabolic pathways significantly varied at each phase, uncovering the non-overlapping niches of the functional groups in the process. Mannose is one of the most abundant mono-saccharides within the *Microcystis* cells (Jürgens et al., 1989). The relative frequency of fructose and mannose metabolism pathways was significantly higher in the treatment group, which suggested that mannose in the biomass could be effectively transformed during the decomposition. Not only that, the enriched amino acid metabolism and secreted peptidases suggested that *Microcystis* biomass could provide plenty of carbon and nitrogen sources for the co-existing microbiomes. Furthermore, the phosphotransferase system plays an important role in the transportation and phosphorylation of numerous mono-saccharides, disaccharides, amino sugars, and other sugar derivatives through the bacterial cell membrane (Deutscher et al., 2006). The higher frequency of the phosphotransferase system in the treatment group indicated the transportation of those small-molecule intermediates following initial extracellular hydrolysis.

In the ecological processes, microorganisms interact with each other and therefore form a complex network within the communities. MENs construction is an ideal way to characterize the species co-occurring pattern in an ecosystem. In general, positive links could be attributed to niche overlap or cross-feeding, and negative links could be attributed to competition or antagonism (Faust and Raes, 2012). In the present study, co-existing taxa in the treatment group was much more connected and clustered than those in the control group, which implied that the decomposition of *Microcystis* or utilization of *Microcystis* substrates required inter-specific cooperation among various functional groups. The lower modularity in treatment group indicated a less resistance of the system to disturbance. Therefore, the enhanced connection in the community might be an adaptive mechanism in response to massive disturbance, i.e., the organic matter impulse.

Taken together, the community dynamics, predicted functions and network analysis reflected the transition of the microbial communities toward a low diverse, high connected and high effective situation for the nutrient assimilation during algal carbon/nitrogen transformation.

## Data Availability Statement

The datasets generated for this study can be found in NCBI, https://www.ncbi.nlm.nih.gov/bioproject/?term=PRJEB21014.

## Author Contributions

Y-FW did the experiment and data analysis. PX provided most of the idea of data treatment and wrote the manuscript. SL and QW did the experiment design and revised the manuscript.

## Conflict of Interest

The authors declare that the research was conducted in the absence of any commercial or financial relationships that could be construed as a potential conflict of interest.

## Footnotes

1 https://www.dsmz.de/microorganisms/medium/pdf/DSMZ_Medium1593.pdf

2 http://picrust.github.com

3 http://huttenhower.sph.harvard.edu/galaxy/

4 http://ieg2.ou.edu/MENA

5 https://www.ncbi.nlm.nih.gov/bioproject/?term=PRJEB21014
