# Supplementary material for: Enhanced Microbial Interactions and Deterministic Successions During Anoxic Decomposition of Microcystis Biomass in Lake Sediment
Source: Front Microbiol. 2019 Oct 30;10:2474. doi: 10.3389/fmicb.2019.02474 (PMC6831559; doi:10.3389/fmicb.2019.02474)

**Enhanced microbial interactions and deterministic successions during anoxic decomposition of *Microcystis* biomass in lake sediment**

Yu-Fan Wu^1, #^, Peng Xing^2, #*^, Shuang-Jiang Liu^1^ and Qing-Long Wu^2, 3, *^

^1^ State Key Laboratory of Microbial Resources, Institute of Microbiology, Chinese Academy of Sciences, Beijing, 100101, P. R. China;

^2^ State Key Laboratory of Lake Science and Environment, Nanjing Institute of Geography& Limnology, Chinese Academy of Sciences, Nanjing 210008, P. R. China

^3^ Sino-Danish Centre for Education and Research, University of Chinese Academy of Sciences, Beijing 100039, China

^4^ Technology Center of Zhangjiagang Customs, Zhangjiagang, P. R. China

^#^ These authors contributed equally to this paper

***Correspondence:**

Prof. Dr. Qinglong L. Wu

[qlwu@niglas.ac.cn](mailto:qlwu@niglas.ac.cn)

or Prof. Dr. Peng Xing

[pxing@niglas.ac.cn](mailto:pxing@niglas.ac.cn)

Running title: Microbial interaction during *Microcystis* decomposition

**Table S1** Primers used for quantitative PCR in this study

| **Primer** | **Sequence (5´-3´)** | **Target** | **Product (bp)** | **Ref** |
| --- | --- | --- | --- | --- |
| 341F  534R | CCTACGGGAGGCAGCAG ATTACCGCGGCTGCTGG | Bacterial 16S rRNA gene | ~190 | (1) |
| Arch 333F  Arch 554R | GAGATGGATTCTGAGACACGAA  TTAGGCCCAATAAAAKCGAC | Archaea 16S rRNA gene (Crenarcheota, Euryarchaeota) | ~106 | (2) |
| ME1  Mlas | GGCGTCGATCTGCGCAAGCC  CAGCGGTTGTCCCACATCAC | Methanogens | ~360 | (3) |

**References**

1. Bru D, Martin-Laurent F, Philippot L (2008) Quantification of the detrimental effect of a single primer-template mismatch by real-time PCR using the 16S rRNA gene as an example. Appl Environ Microbiol 74:1660-1663. doi:10.1128/AEM.02403-0

2. Suzuki MT, Taylor LT, Delong EF (2000) Quantitative analysis of small-subunit rRNA genes in mixed microbial populations. Appl Environ Microbiol 66:4605-4614.

3. Steinberg LM, Regan JM (2008) Phylogenetic comparison of the methanogenic communities from an acidic, oligotrophic fen and an anaerobic digester treating municipal wastewater sludge. Appl Environ Microbiol 74: 6663-6671. doi: 10.1128/AEM.00553-08

**Fig. S1** The differences of microbial community structures between day 0 and the other time points based on Bray-Curtis distance. The line chart indicated that the addition of *Microcystis* biomass and its decomposition have dramatically changed the microbial structures, when compared to the Control.

**
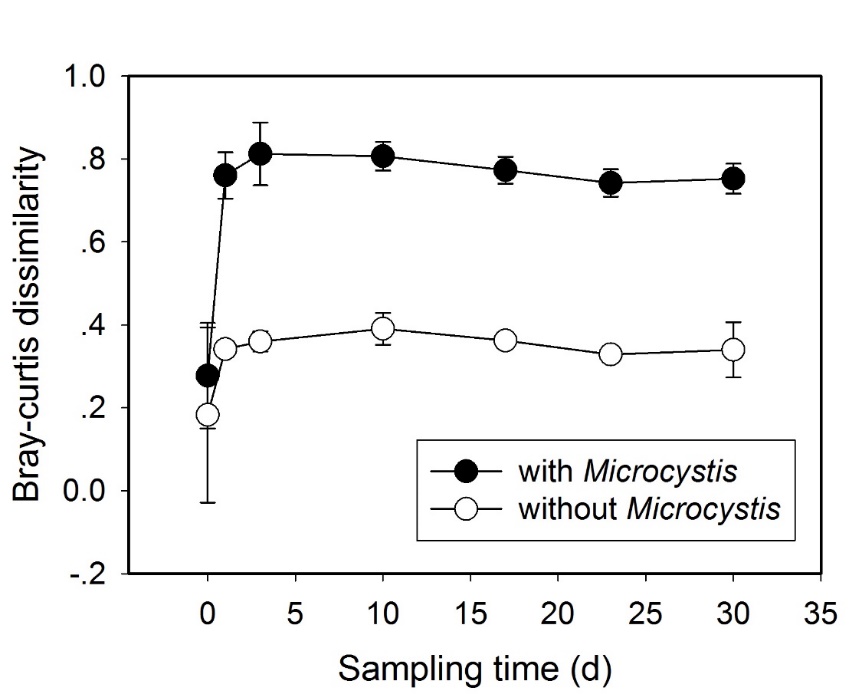
**

**Fig. S2** Microbial community composition of the microcosm samples at phylum level. Each bar represented the average relative abundance of biological replicates for a certain bacterial phylum. The top 20 abundant phyla were shown here.

**Fig. S3** The relative contribution of methanogen to the whole sequences in each sample during *Microcystis* decomposition. Eight methanogenic genera were detected in the microcosm during the incubation. Two most abundant genera, *Methanosarcina* and *Methanobacteriaceae* unidentified species, increased from the beginning and peaked at day 17 then followed a slightly decrease after that.

**Fig. S4** The KEGG subpathway-II associated with each of the nine subpathway-I (significantly activated by the *Microcystis* addition, shown in Fig. 6). Extended error plots for functional gene predications by STAMP using KEGG subpathway I (the lowest classification group) with a two-sided Welch’s *t*-test of a symptomatic confidence intervals (0.95). Subpathways overrepresented in the Treatment which have a positive value of proportions are indicated by orange filled circles and those overrepresented in the Control are indicated by blue filled circles.

1. Membrane Transport


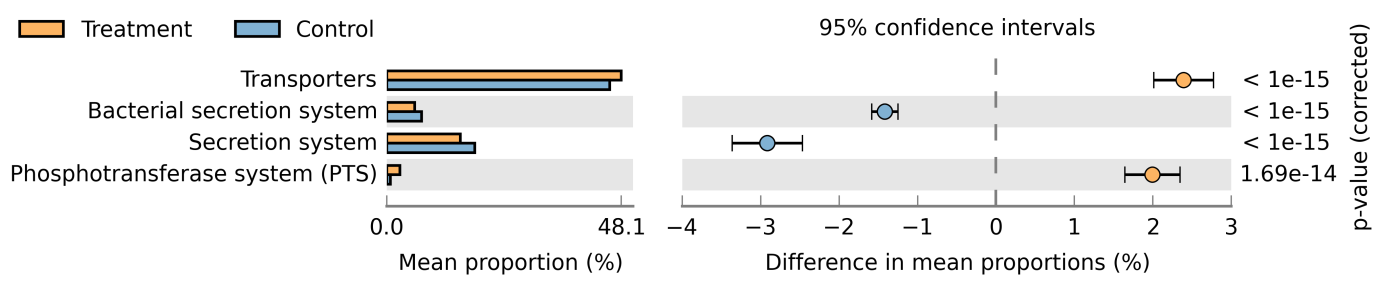


1. Replication and Repair


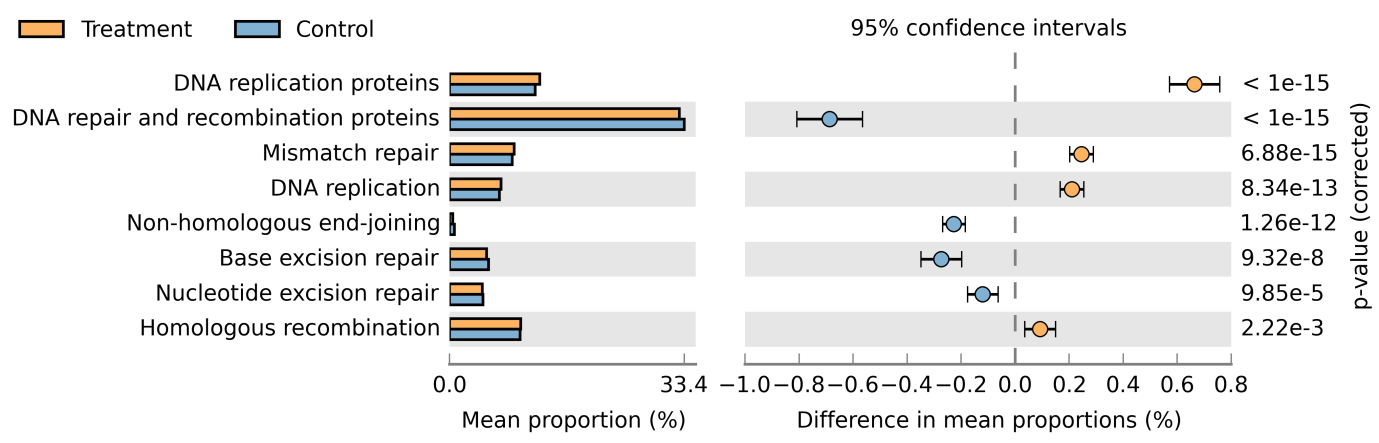


1. Carbohydrate Metabolism


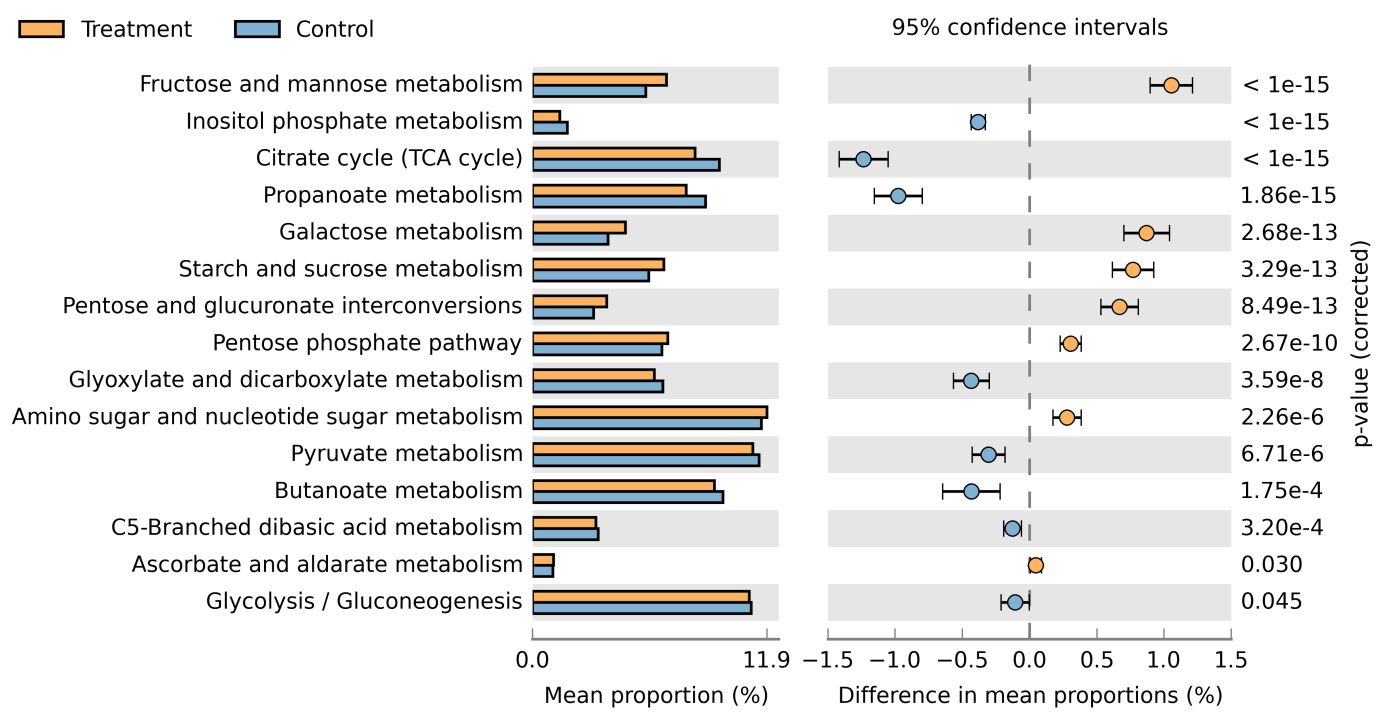


1. Amino Acid Metabolism


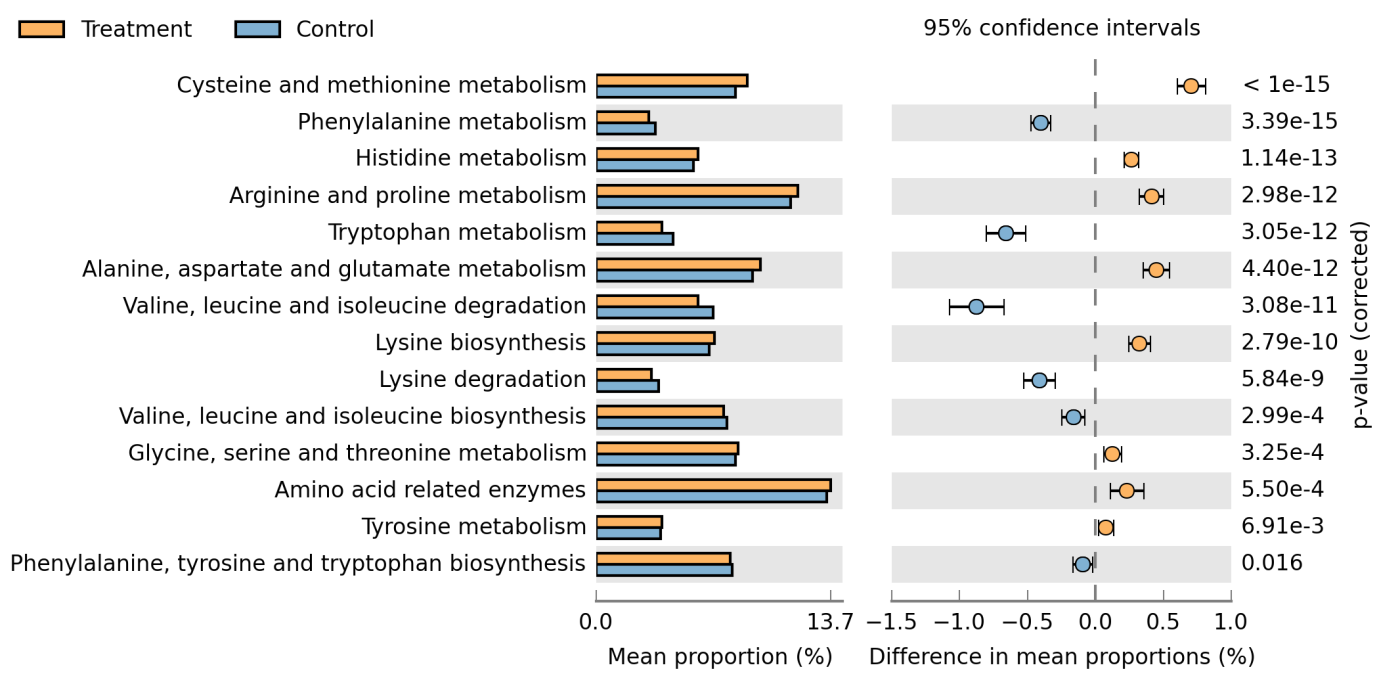


1. Transcription


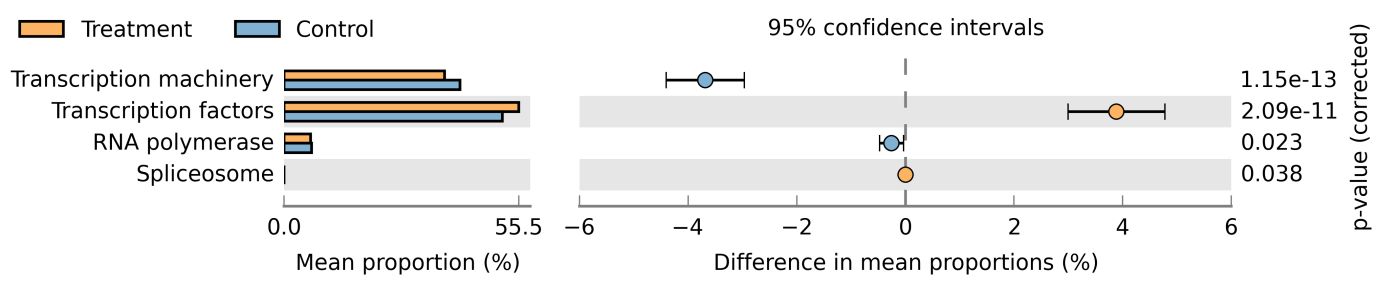


1. Metabolism of cofactors and vitamins


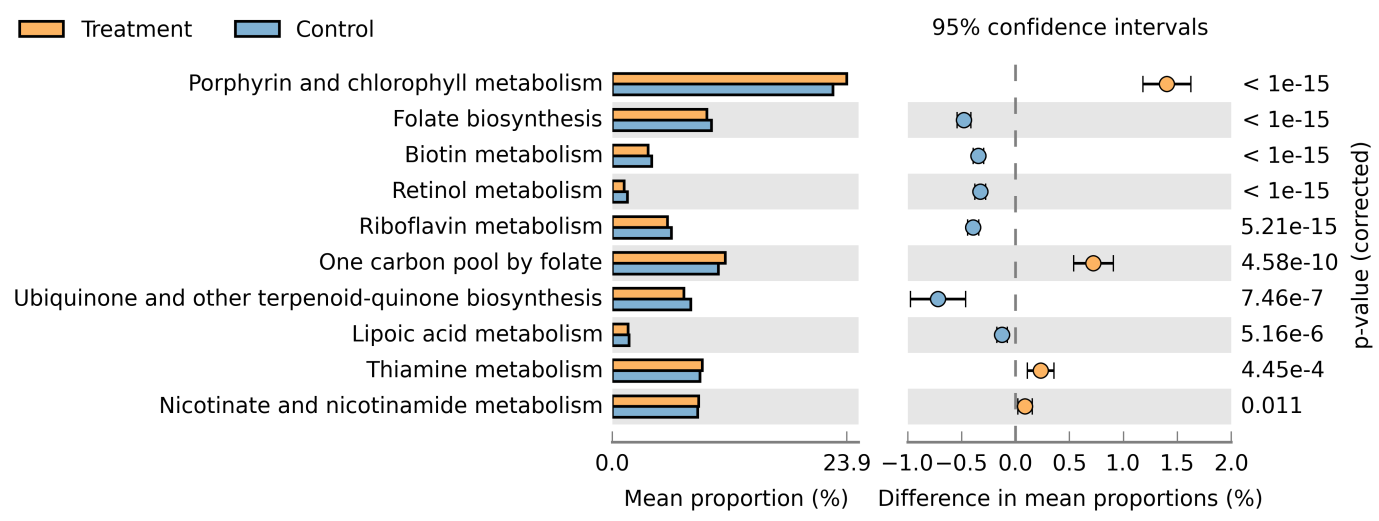


1. Nucleotide Metabolism


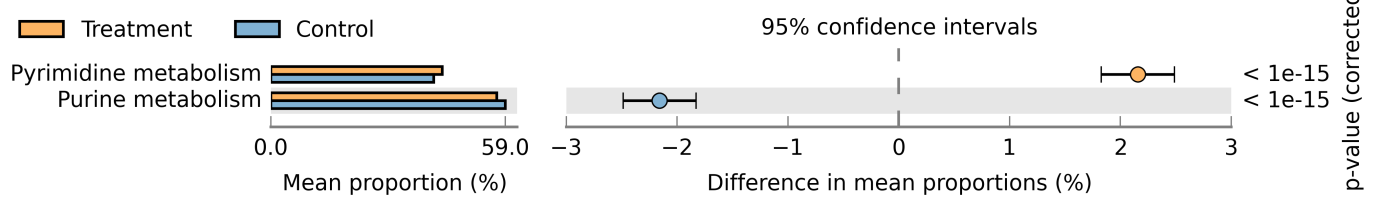


1. Enzyme Families


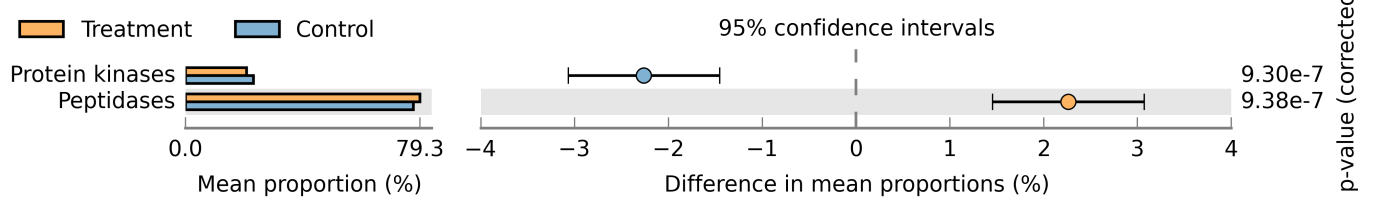


1. Metabolism of Other Amino Acids


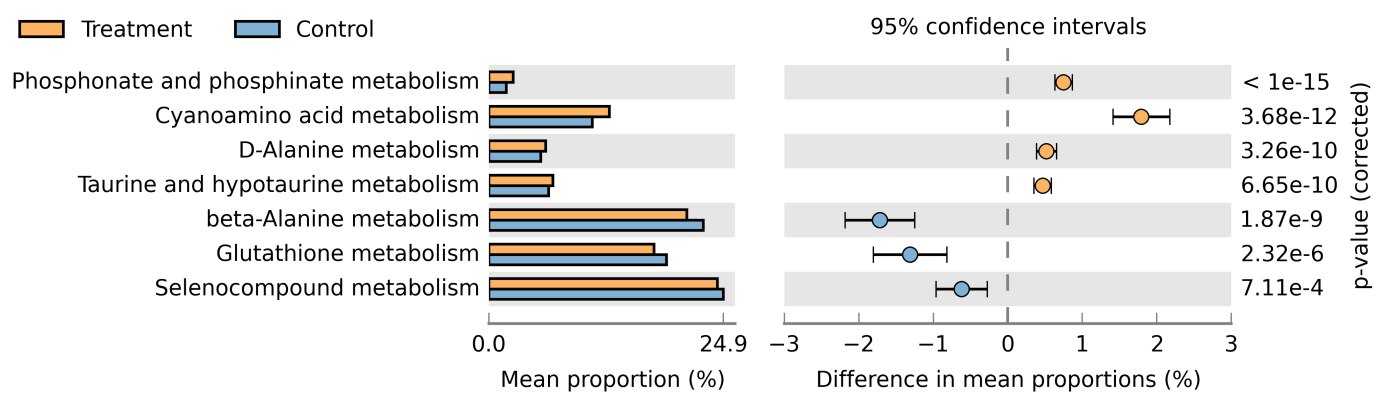

Supplement: Supplementary file 1 [file Data_Sheet_1.docx]
